# Hippocampal Fast Glutamatergic Transmission Is Transiently Regulated by Corticosterone Pulsatility

**DOI:** 10.1371/journal.pone.0145858

**Published:** 2016-01-07

**Authors:** R. Angela Sarabdjitsingh, Natasha Pasricha, Johanna A. S. Smeets, Amber Kerkhofs, Lenka Mikasova, Henk Karst, Laurent Groc, Marian Joëls

**Affiliations:** 1 Dept. Translational Neuroscience, Brain Center Rudolf Magnus, University Medical Center Utrecht, Utrecht, The Netherlands; 2 Universite de Bordeaux, Interdisciplinary Institute for Neuroscience, UMR 5297, F-33000 Bordeaux, France; 3 CNRS, IINS UMR 5297, Bordeaux, France; IIBB/CSIC/IDIBAPS, SPAIN

## Abstract

In recent years it has become clear that corticosteroid hormones (such as corticosterone) are released in ultradian pulses as a natural consequence of pituitary-adrenal interactions. All organs, including the brain, are thus exposed to pulsatile changes in corticosteroid hormone level, important to ensure full genomic responsiveness to stress-induced surges. However, corticosterone also changes neuronal excitability through rapid non-genomic pathways, particularly in the hippocampus. Potentially, background excitability of hippocampal neurons could thus be changed by pulsatile exposure to corticosteroids. It is currently unknown, though, how neuronal activity alters during a sequence of corticosterone pulses. To test this, hippocampal cells were exposed *in vitro* to four consecutive corticosterone pulses with a 60 min inter-pulse interval. During the pulses we examined four features of hippocampal signal transfer by the main excitatory transmitter glutamate—i.e., postsynaptic responses to spontaneous release of presynaptic vesicles, postsynaptic GluA2-AMPA receptor dynamics, basal (evoked) field responses, and synaptic plasticity, using a set of high resolution imaging and electrophysiological approaches. We show that the first pulse of corticosterone causes a transient increase in miniature EPSC frequency, AMPA receptor trafficking and synaptic plasticity, while basal evoked field responses are unaffected. This pattern is not maintained during subsequent applications: responses become more variable, attenuate or even reverse over time, albeit with different kinetics for the various experimental endpoints. This may indicate that the beneficial effect of ultradian pulses on transcriptional regulation in the hippocampus is not consistently accompanied by short-term perturbations in background excitability. In general, this could be interpreted as a means to keep hippocampal neurons responsive to incoming signals related to environmental challenges.

## Introduction

The hypothalamic-pituitary-adrenal (HPA) axis, rather than being in a static state, is continuously and dynamically equilibrated by rhythmic release of corticosteroids throughout the day [1]. In mammals, this secretory pattern by the adrenal gland typically results in rapidly increasing and decreasing levels of corticosteroids in blood plasma (cortisol in humans, corticosterone in rodents) with a periodicity of approximately 60 min. These ultradian corticosteroid pulses decrease in amplitude during the active phase and increase during the inactive phase, which shapes the characteristic circadian profile of hormone release [2,3]. The significance of ultradian pulsatility is just starting to be understood [1]. The resilience in the neuroendocrine and behavioral response to stress is essentially coordinated by the timing and pattern of glucocorticoid release [4–8]; for instance, constant administration of corticosterone causes a blunted ACTH response to a noise stressor, while pulsatile exposure to corticosterone results in a stronger response to stress administered during the rising phase than the falling phase of the pulse [5].

Ultradian corticosterone pulses persist in target tissues, such as the brain, where the steroid receptors reside [9,10]. In the hippocampus, an essential area for learning and memory, both the mineralocorticoid (MR) and glucocorticoid receptor (GR) function as ligand-activated transcription factors to modulate genomic events via transcriptional activation or repression of target genes [11,12]. Accumulating evidence now points towards an important role of ultradian pulses in the strength of GR signaling, enabling a transcriptionally efficient response to stress and preventing receptor desensitization, such as occurs during constant corticosterone administration [13–16]. Accordingly, we recently showed that ultradian pulsatility is necessary to balance the delayed (genomic) corticosteroid effects on hippocampal glutamatergic neurotransmission and synaptic plasticity [17].

Next to these transcriptional effects on brain functioning, corticosteroids can exert rapid non-genomic actions [18]. In the hippocampus, rapid corticosterone effects were found to be mediated by membrane located MRs, causing an increased frequency in AMPA receptor-mediated synaptic currents [19,20], as well as decreased potassium A-currents and enhanced AMPA receptor mobility [21–23]. Additionally, synaptic plasticity was shown to be rapidly facilitated when corticosterone and high-frequency stimulation coincide in time [24]. Collectively, these studies investigating hippocampal properties during a single pulse of corticosterone point to facilitating effects on glutamatergic neurotransmission and synaptic plasticity via rapid corticosteroid signaling pathways.

Potentially, this non-genomic pathway may quickly translate natural shifts in corticosteroid level–such as occur during ultradian pulses- into changes in hippocampal glutamatergic signaling and thus affect the stability of information transfer. However, whether this translation occurs is uncertain, because non-genomic responses are liable to metaplasticity [9,25] and/or over time could be modulated by gradually developing genomic effects. Therefore, we here examined how hippocampal glutamatergic transmission and synaptic plasticity are affected *during* a sequence of hourly corticosterone pulses; this complements earlier studies where glutamatergic transmission and synaptic plasticity were examined in the periods *in-between* pulses [17]. We presently investigated four aspects of glutamatergic transmission reflecting *i*) presynaptic glutamate release, *ii*) postsynaptic AMPA receptor dynamics, and *iii*) postsynaptic field responses and iv) long-term potentiation (LTP) *during* four brief corticosterone pulses administered with a 1 hr interpulse interval to hippocampal cultured cells or acutely prepared brain slices.

## Materials and Methods

### Experimental design

To study the effect of repetitive corticosterone application on AMPA receptor trafficking, miniature excitatory postsynaptic current (mEPSC) properties and synaptic plasticity, 10 min pulses of corticosterone (100 nM, Sigma-Aldrich) or vehicle (0.09% ethanol) were applied to hippocampal cultured cells or acutely prepared mouse brain slices containing the hippocampus. Tissue was exposed to 1 to 4 consecutive pulses with an interpulse interval of one hour. The selected paradigm has been used in literature before and effectively resulted in a pulsatile pattern, with an overall pulse-width of approximately 20 min [9,17].

### Primary hippocampal cell culture

As previously described [21], cultures of hippocampal neurons were prepared from the brains of 18 day old Sprague-Dawley rat embryos. Briefly, cells were plated at a density of 200–300 10^3^ per dish and grown on poly-L-lysine-coated coverslips. Mixed cultures of neurons and glial cells were layered on coverslips and maintained in a 3% serum containing neurobasal medium. This medium was replaced after 4 days in vitro (DIV) by a serum-free neurobasal medium. A HEPES buffer was used as medium for the experiments (in mM):140 NaCl, 5 KCl, 2.5 CaCl_2_, 1.6 MgCl_2_, 10 Hepes and 24 D-glucose. Cultures were maintained at 37°C in 5% CO2.

### Single Quantum Dot tracking

Quantum dot (QD) experiments for GluA2 receptor trafficking were performed in hippocampal neuronal cultures (14–21 DIV). For each experiment, 3 to 6 sets of cultures were used. As previously described [26,27], cultured hippocampal neurons were incubated at 37°C for ten minutes with a GluA2- mouse antibody (1:800, Millipore). After washing with warm medium, cells were incubated at 37°C for ten minutes with a mix of QD anti-mouse 655 (1:1000, Invitrogen) and casein, to prevent nonspecific binding (1:1000, Vector Laboratories, France). Incubation with Mitotracker (1:10000, Green Mitotracker, Molecular Probes) was applied for later detection of synapses. QDs were visualized using a custom wide-field single-molecule fluorescence inverted microscope (x100 oil immersion objective). Recording was performed by an EMCCD camera; image acquisition was performed using MetaMorph (Universal imaging Corp). All recording sessions were acquired within 30 min following primary antibody incubation to minimize receptor endocytosis. Twenty frames (50 ms per frame) were recorded with the transmission light and averaged to have a clear overall picture of the neuronal structure. Synapses were visualized by using Green Mitotracker and appropriate emission/excitation filters; here again, twenty frames (50 ms per frame) were recorded and averaged. QDs were detected by using a xenon lamp (560RDF55, Omega) and appropriate emission filters (655WB20; Omega Filters). QD-labeledGluA2 subunits were followed in randomly selected dendritic regions for up to 20 min. To track the receptor dynamics, a thousand frames were recorded (50 ms per frame) and saved as a stack file. Two movies were recorded prior to giving the pulse (for baseline recording) and two movies were recorded during the last 5 minutes of the rising phase of a corticosterone pulse. Recorded trajectories of single molecules were reconstructed by correlation analysis between consecutive images, using a Vogel algorithm. Single QD particles were continuously tracked. Non-specifically bound QDs were removed from the analysis. The instantaneous diffusion coefficient, D, was calculated for each trajectory, from linear fits of the first 4 points of the mean-square-displacement versus time function using MSD(t) = <r2> (t) = 4Dt.

### Animals for electrophysiology

Male C57BL/6 mice (Harlan, The Netherlands, approx. 5–6 weeks of age at arrival) were group-housed at a 12-h light-dark schedule (lights on at 07.00 AM) with food and water provided ad libitum. After an acclimatization period of approximately 1–2 weeks, mice (one at a time) entered the experiment. Mice were decapitated early in the morning when endogenously circulating plasma corticosterone levels are still low. All experiments were approved by the Animal Ethical Committee from Utrecht University and all efforts were made to minimize suffering of the animals.

### Electrophysiological recordings

After decapitation, the mouse brain was rapidly dissected and placed in ice-cold aCSF (artificial cerebrospinal fluid) containing (in mM): 120 NaCl, 3.5 KCl, 1.3 MgSO_4_, 1.25 NaH2PO_4_, 2.5 CaCl_2_, 10 glucose and 25 NaHCO_3_ and continuously gassed (mixture of 95% O_2_-5% CO_2_). Next, dorsal hippocampal slices (350 μm) were made using a vibratome (LEICA VT 1000S) and stored in aCSF at room temperature for >1h before recording commenced at a bath temperature of 30–32°C [19].

Miniature excitatory postsynaptic currents (mEPSCs) from CA1 pyramidal neurons in brain slices were recorded with patch clamp electrophysiology as previously described [19,20]. One slice at a time was transferred to the recording chamber mounted on an upright microscope (Axioskop 2 FS plus, Zeiss, Oberkochen, Germany) with differential interference contrast and a water immersion objective (x40) to identify the cells. Slices were continuously perfused (flow rate 1.5 ml/min) with aCSF containing TTX (0.5 μM; Latoxan, France) to block sodium channels and bicuculline (50 μM; Enzo) to block GABAa receptors. Patch pipettes (tip resistance: 3–6 MΩ) were filled with the pipette (intracellular) solution containing (in mM): 120 Cs methane sulfonate, 17.5 CsCl, 10 Hepes, 2 MgATP, 0.1 NaGTP, 5 BAPTA (Molecular Probes, the Netherlands); pH 7.4.An Axopatch 200B amplifier (Axon Instruments, USA) was used for whole cell recordings, operating in the voltage-clamp mode at a holding potential of -70 mV. The liquid junction potential caused a shift of 8 mV at most for which we did not compensate. Recordings with an uncompensated series resistance of <2.5 times the pipette resistance were accepted for analysis. In view of the small current amplitudes, the recordings were not corrected for series resistance.

Currents were identified as mEPSCs when the rise time was faster than the decay time. Approximately 10–15 min after establishing the whole-cell configuration, mEPSCs were recorded under baseline conditions, followed by recording during 10minutes application of corticosterone (100 nM) and 10 minutes after washout. In this study we were primarily interested in the relative changes in mEPSC properties over the four pulses of corticosterone, comparing responses to each of the pulses of corticosterone, i.e. the final 5 min of corticosterone application, to the 5 min of baseline just preceding the respective pulse (1st, 2nd, 3rd, 4th pulse). As earlier experiments indicated that vehicle application is entirely ineffective in changing mEPSC properties after one pulse [19], we restricted control experiments to only examining if neurons were affected by exposure to a total of 4 consecutive pulses of vehicle. Data was analyzed offline using ClampFit 9.2 as previously described [19]. For each cell the frequency, amplitude, tau of rise time and tau of decay of mEPSCs were determined. We here only report on mEPSC frequency and amplitude.

Field Excitatory Postsynaptic Potentials (fEPSPs) were recorded in the Schaffer Collateral-CA1 pathway as described previously [24,28]. Briefly, a bipolar stimulation electrode (60 μm stainless-steel wires insulated except for the tip) was placed on the Schaffer collaterals and glass recording pipettes (filled with aCSF; 2–5MΩ impedance) were positioned in the CA1 stratum radiatum. At the start of each experiment, an input–output curve was established to record the slope of the fEPSP, from which maximal and half-maximal slope as well as the corresponding maximal and half-maximal stimulus intensity were determined. The half maximal stimulus intensity that was thus calculated was used throughout the remainder of the recording session. For each experimental group, baseline synaptic transmission was recorded with a frequency of 0.033 Hz (0.15 ms duration) for 10 min, after which 100 nM corticosterone in aCSF was perfused onto the sections for another 10 min (n = 6–9). At the end of this 10 min application period, repetitive tetanic stimulations (10 Hz; 90 s) were applied, after which recordings proceeded for another 60 min at a frequency of 0.033 Hz; this stimulation paradigm is very sensitive to the effects of corticosterone [24]. Two consecutive traces were averaged to represent the mean per minute. Data were acquired, stored, and analyzed off-line using Signal 2.16 (Cambridge 159 Electronic Design, United Kingdom).

### Statistical analysis

All statistical analyses were carried out with SPSS 21.0. Group values are expressed as mean ± SEM. The relative changes in mEPSC properties were analyzed for each corticosterone pulse (compared to the baseline just before hormone application), with a two-tailed paired Student’s *t* test. Both baseline mEPSC properties prior to each of the four pulses of corticosterone and gradual shifts in the responsiveness to corticosterone over the four pulses are considered to be independent data points. Therefore we applied a one-way ANOVA, followed by Tukey’s post hoc analysis. The results were highly comparable to a separately run mixed model analysis, and the latter is therefore not explicitly mentioned in the text.

For the QD experiments, comparisons between groups for instantaneous diffusion coefficients were performed using non-parametric statistical tests, Mann Whitney test (pair comparison) or Kruskal-Wallis followed by Dunn’s Multiple Comparison Test (group comparison). A mixed model with two factors (treatment, time) was used for between-group comparisons of time-dependent effects of corticosterone. Comparisons between before-after conditions in different pulses for synaptic dwell time and synaptic content were performed using a non-parametric Mann Whitney test or Kruskal-Wallis statistical test.

For the fEPSP recordings, a three-way mixed design ANOVA (treatment, time and infusion) was used to assess whether differences existed between the slope of the baseline recording before and during the infusion of a single, two, three or four corticosterone or vehicle pulses. Student’s paired t-tests were used to analyze within each treatment group whether high frequency stimulation induced significant potentiation compared to the pre-tetanus baseline values. A two-way ANOVA was used to test between treatment groups for possible differences in LTP due to the number of pulses administered or vehicle/corticosterone treatment. Where applicable, Tukey’s post-hoc tests were used. P values < 0.05 were considered significantly different.

## Results

### The effect of corticosterone pulses on CA1 mEPSC properties

We first investigated the impact of corticosterone pulsatility on CA1 hippocampal mEPSC frequency, a presynaptic property, which was previously reported to be quickly enhanced by corticosterone [19].

**Fig 1:**
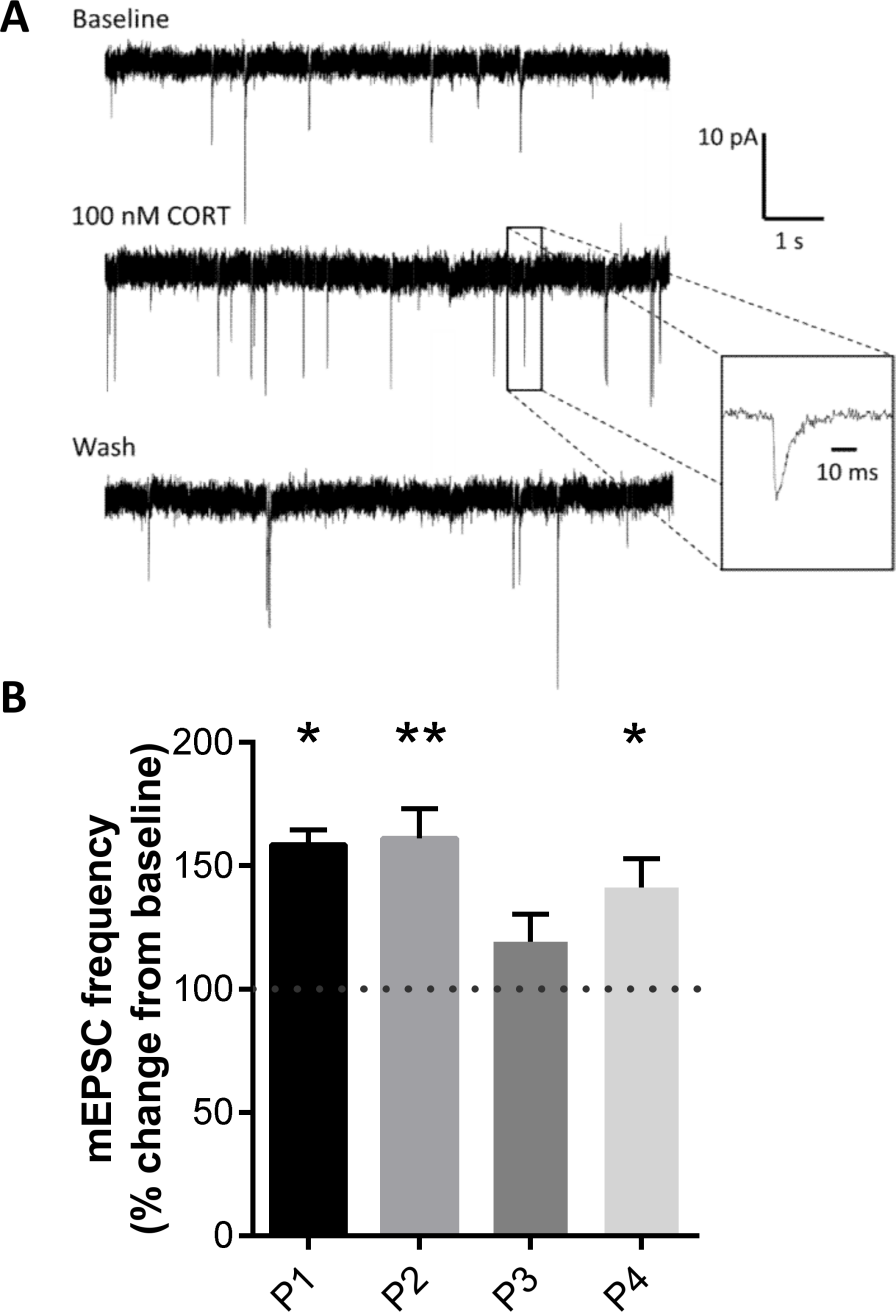
Corticosterone-induced changes in mEPSC frequency (transiently) attenuate. (A)Experimental design. Corticosterone (CORT, 100 nM) or its vehicle was applied for 10 min (thin horizontal arrow), followed by 50 min application of the standard recording buffer. This sequence was repeated four times. For each pulse, the mEPSC properties were recorded during the final 5 min of CORT application (bold vertical arrow) and compared with the properties recorded in the 5 min just prior to hormone application. Statistical analysis is described in the Methods section. A highly similar design was used to determine changes in GluA2 subunit properties during four pulses of CORT; for each pulse this was compared to the values observed just prior to CORT application (see Fig 2). (B) Typical mEPSC trace before, during and 10 min after the first corticosterone application, in an identified CA1 pyramidal cell. An example of a single mEPSC with higher time resolution is shown in the inset. (C) Relative change in mEPSC frequency compared to the pre-pulse baseline (= 100%) after each of four pulses of corticosterone. Data show the mean ± SEM change (n = 6 cells for each pulse). Statistically significant changes in mEPSC frequency due to application of corticosterone (compared to baseline) are indicated by * (p<0.05) or ** (p<0.01).

Pyramidal CA1 neurons in mouse hippocampal slices responded rapidly to a brief (10 min; Fig 1A) pulse of corticosterone in a comparable manner as observed earlier with a 20 min pulse [19]: the mEPSC frequency was quickly elevated and returned to baseline after washout of the hormone (Fig 1B). On average, the frequency in the final 5 minutes of the first corticosterone application was significantly enhanced compared to the baseline in the 5-min period just prior to hormone application (Fig 1C; p < 0.05). Similar results were observed after a second pulse application one hour later (Fig 1C; p < 0.01). Responses to a third pulse were attenuated (p = 0.39). During the 4th application of the hormone mESPC responsiveness to corticosterone was partially restored (p = 0.04). Interestingly, baseline mEPSC frequency before each pulse remained comparable over time (F_(3,20)_ = 0.6; p = 0.62, Table 1). Earlier, it was shown that exposure of hippocampal CA1 neurons to a single pulse of vehicle did not result in a change in mEPSC frequency [19]. In the present study, we additionally observed that exposure to multiple consecutive pulses of vehicle was equally ineffective in changing mEPSC frequency (0.32 ± 0.07 Hz, n = 7 prior to the fourth pulse of vehicle; and 0.34 ± 0.05 Hz, n = 7 during the fourth pulse of vehicle; p = 0.70). The mEPSC amplitude was unaffected over time, both at baseline (F_(3,20)_ = 0.62, p = 0.61; Table 1) and with respect to the relative change in amplitude during corticosterone application (F_(3,20)_ = 2.2, p = 0.12; Table 1).

**Table 1:** Absolute baseline mEPSC frequency and amplitude (± SEM) recorded in CA1 pyramidal cells for each experimental group. Changes during each pulse of corticosterone (% relative to the corresponding baseline) are only shown with respect to the amplitude; for relative changes in mEPSC frequency we refer to Fig 1. Based on 6 cells for each experimental group.

mEPSC properties in CA1 pyramical cells	P1	P2	P3	P4
**Baseline frequency (Hz)**	0.70 ± 0.09	0.56 ± 0.11	0.56 ± 0.11	0.52 ± 0.04
**Baseline amplitude (pA)**	17.6 ± 1.1	15.3 ± 0.7	15.4 ± 2.0	15.8 ± 1.9
**Change in amplitude (%)**	-2.1 ± 5.7	-9.1 ± 10.9	18.0 ± 6.2	10.7 ± 10.4

Altogether these data indicate that upon repetitive application of corticosterone pulses, CA1 pyramidal mEPSC frequency is most clearly affected by the first two pulses and less so by subsequent pulses, without affecting the pre-pulse baseline.

The effect of corticosterone pulses on GluA2 receptor subunit surface diffusion

**Fig 2:**
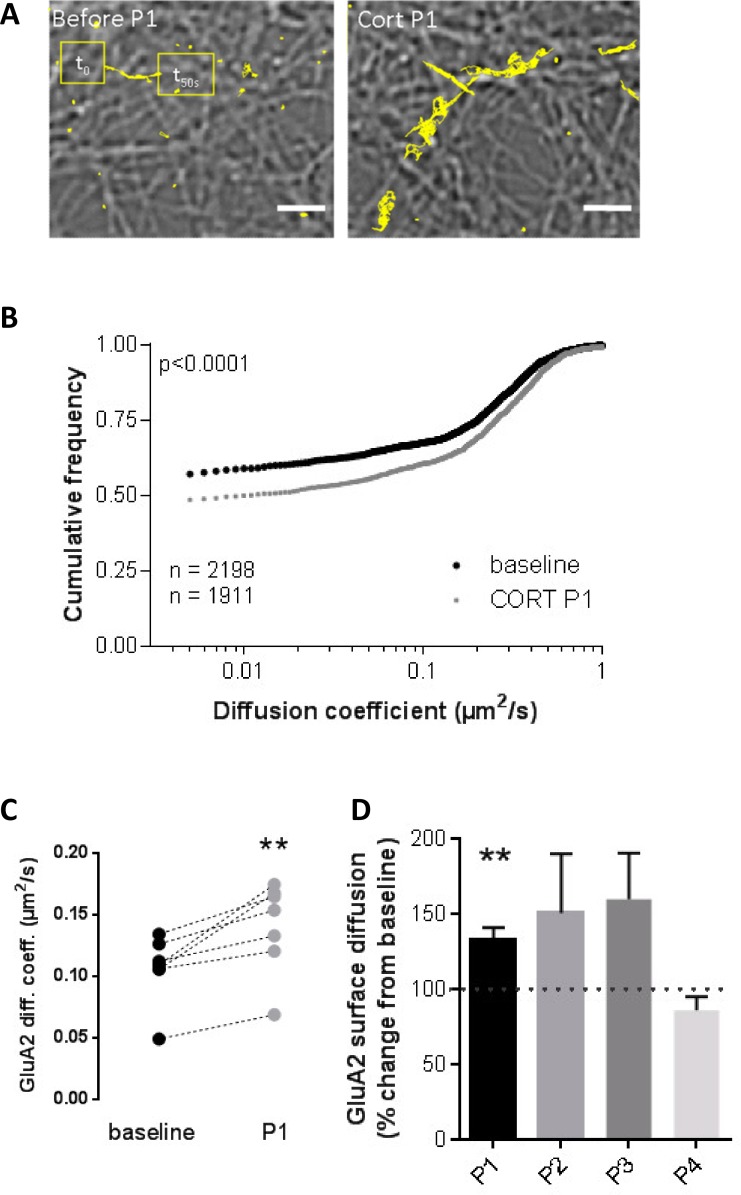
Consecutive corticosterone pulses differentially influence GluA2-AMPAR trafficking. (A) Representative trajectory (10–50 s duration) of surface GluA2 before and after the first pulse of corticosterone (peak at 100 nM). Scale bars represent 5 μm. (B) The total diffusion coefficient was significantly increased after the first pulse of corticosterone (cumulative frequency, n = 2198 for control and n = 1911 for corticosterone). (C) The first corticosterone pulse (peak at 100 nM) significantly increased the mean surface diffusion of the GluA2 receptor (mean, n = 7). (D) The corticosterone-induced increase in GluA2 surface diffusion was only observed after the first pulse (bars represent mean ± SEM, n = 1441–2445 trajectories per group, n = 7 experiments). Changes were much more variable (and non-significant) during pulse 2 and 3; no change at all was observed during the 4^th^ pulse. ** P<0.01.

As a next step, we examined a postsynaptic index of glutamatergic transmission which was previously found to be rapidly affected by corticosterone: the mobility of GluA2-containing AMPARs; this was found to be quickly and reversibly enhanced by a single pulse of corticosterone [21]. We used single nanoparticle tracking to monitor individual particle/receptor complexes in primary hippocampal cultures (Fig 2).

Consistent with earlier results [21], synaptic trafficking of the GluA2-AMPAR was significantly increased during the peak of the first corticosterone pulse (Fig 2A–2C; p < 0.01), compared to the baseline. Consecutive corticosterone pulses however differentially affected GluA2 receptor trafficking (Fig 2D; mixed model ANOVA, interaction effect time x treatment: F_(1, 3)_ = 5.6; p < 0.001). Follow-up analysis revealed that a gradual attenuation in surface diffusion occurred during subsequent pulses. While a trend towards increased GluA2 surface diffusion was observed during the 2nd and 3rd pulse, responses were much more variable. We observed no effect during the 4th pulse. The total number of molecules tracked did not change during the whole experiment (Kruskal-Wallis comparing medians, p = 0.1548), indicating that the observed differences between diffusion coefficients after corticosterone application were not due to overall changes in GluA2 receptor number. Baseline values remained stable over time (F_(3, 24)_ = 0.62; p = 0.61; Table 2). A single pulse of vehicle (HEPES) did not affect GluA2 dynamics (GluA2 surface diffusion: absolute mean ± SEM μm/s; baseline 0.1494 ± 0.008 μm/s, N = 371 vs 0.1547 ± 0.01 μm/s, N = 356; p = 0.48), nor was any effect of vehicle seen during subsequent pulses, including the fourth consecutive pulses of HEPES (baseline 0.1011 ± 0.011 μm/s, N = 173 vs 0.079 ± 0.011 μm/s, N = 153; p = 0.079 one-way ANOVA, Dunn’s multiple comparison test, p>0.05).

**Table 2:** Absolute values of surface GluA2 AMPA instantaneous diffusion coefficients prior to the 4 corticosterone pulses. The diffusion coefficient is expressed in μm^2^/s (see Methods for calculation) and was obtained in live cultured hippocampal neurons of at least 10 days *in vitro*.

GluA2 total diffusion parameters	P1	P2	P3	P4
**Number of trajectories (N)**	2198	2445	1837	1531
**Median (μm^2^/s)**	0.00154	0.00175	0.00194	0.00088
**Inter-quartile range (μm^2^/s)**	0.000368–0.2024	0.00038–0.17065	0.000443–0.1022	0.000284–0.0301
**Mean ± SEM (μm^2^/s)**	0.1125 ± 0.0039	0.1052 ± 0.0036	0.0837 ± 0.0038	0.0864 ± 0.0045

Corticosterone also resulted in a significantly shorter dwell time of GluA2-AMPAR in the synaptic area, but only during the first two pulses (unpaired t test, p<0.0001, Table 3). We then compared the fraction of synaptic GluA2-AMPAR QD at different times and pulse positions. No significant difference between pulses was observed (2-way ANOVA, Factor Pulse: F_(1,3)_ = 1.97, p = 0.12; Factor Corticosterone F_(1,3)_ = 0.37, p = 0.54). Consistently, we report no change of the synaptic GluA2-AMPAR fraction for any of the pulses (i.e. each pulse is compared to its own baseline; pulse 1: p = 0.47, pulse 2: p = 0.45, pulse3: p = 0.91, pulse 4: p = 0.62, Mann-Whitney test).

**Table 3:** Values of GluA2 AMPAR synaptic dwell-time: before (baseline) and during corticosterone exposure. The values are expressed in seconds and as (mean ± SEM). The synaptic dwell-time corresponds to the time (sec) spent by a diffusing GluA2-AMPAR within the synaptic area (see Methods for details). The values were obtained in live cultured hippocampal neurons of at least 10 days *in vitro*.

GluA2 synaptic dwell time	P1	P2	P3	P4
**Baseline**	0.475 ± 0.025	0.481 ± 0.036	0.358 ± 0.024	0.4365 ± 0.042
**During corticosterone**	0.318 ± 0.018	0.373 ± 0.021	0.421 ± 0.032	0.441 ± 0.038

Altogether, these results indicate that GluA2-AMPAR surface trafficking in hippocampal neurons is particularly responsive to the first pulse of corticosterone, less consistently to the 2^nd^ and 3^rd^ pulse and insensitive to the 4^th^ pulse.

### The effect of corticosterone pulses on baseline fEPSP responses

**Fig 3:**
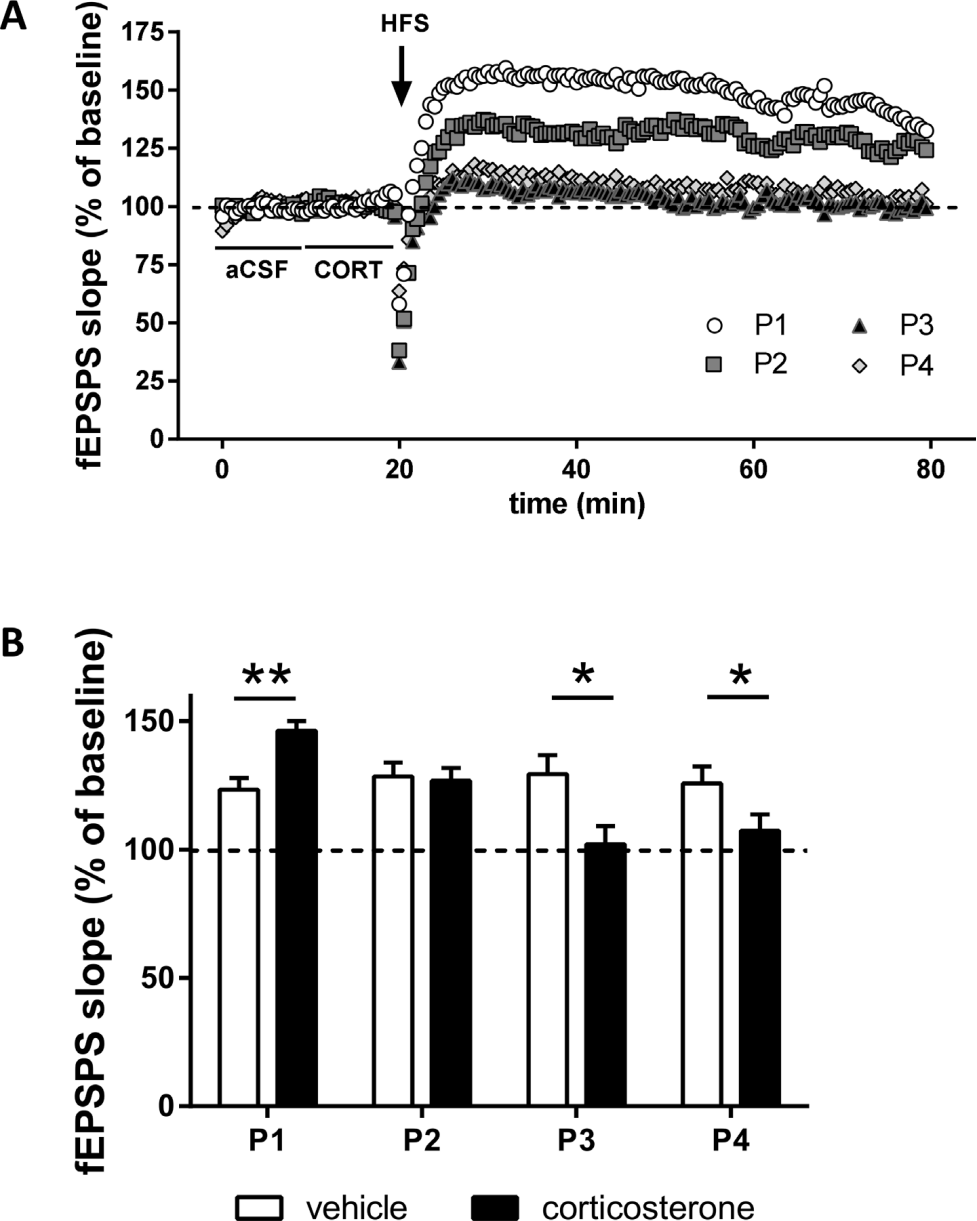
Consecutive corticosterone application attenuates hippocampal LTP. (A) Experimental design. Corticosterone (CORT, 100 nM) or its vehicle was applied for 10 min (thin horizontal arrow), followed by 50 min application of the standard recording buffer. This sequence was repeated four times. Baseline fEPSP properties were recorded 10 min prior to each pulse, during the pulse and up to 50 minutes after the pulse (bold vertical arrow). The fEPSP slope measured at this time point was compared with that recorded prior to the pulse and the relative changes are depicted in part C. Coinciding with the end of the CORT or vehicle pulse, high frequency stimulation (HFS) was applied to the slice (lightning bolt). HFS was delivered only once in each slice. Statistical analysis is described in the Methods section. (B) HFS (high-frequency stimulation) resulted in significant potentiation of synaptic responses, as seen in CA1 synapses of brain slices exposed to a corticosterone pulse (open circles). HFS applied during subsequent pulses resulted in attenuated LTP (P2 = grey squares, P3 = black triangles; P4 = grey diamonds; n = 7–9 for each pulse). Data represent mean ± SEM. (C) Averaged mean values during the 60-min post-tetanic recording period indicate the gradual attenuation in LTP after consecutive corticosterone pulses (black bars), while vehicle treatment did not affect synaptic plasticity over time (open bars). Values indicate group means ± SEM (** P < 0.01; * P < 0.05; n = 6–9 per group).

Corticosterone-induced changes in pre- and postsynaptic glutamate signaling may be reflected in altered responses of fields of neurons. To investigate whether the infusion of corticosterone (or vehicle) pulses acutely affected field responses, we analyzed the averaged responses of the first 10 min (t = 0 to 10 min; Fig 3A) during baseline (perfusion with aCSF) and compared this with averaged basal responses while corticosterone was administered (t = 10 to 20 min; Table 4, Fig 3B (0–20 min)). No effect of corticosterone infusion on baseline fEPSP slope was found (p = 0.14). This analysis was also carried out for baseline recording prior to the 2^nd^, 3^rd^ and 4^th^ pulse, using a mixed model ANOVA with the factors treatment (vehicle vs corticosterone) and number of pulses (one, two, three or four pulses) as between-factors and time (baseline before vs during corticosterone / vehicle administration) as within-factor. No main between-subject effects of treatment (F_(1,50)_ = 0.016, p = 0.90) or number of pulses was found (F_(3,50)_ = 0.26, p = 0.85), nor did we find any significant interaction effect (pulse x treatment (F_(3,50)_ = 1.66; p = 0.19). A trend for within-subject factor time was found (F_(1,50)_ = 3.91; p = 0.054) though no interaction effect was found with either treatment, pulse or both. Overall, these findings suggest that regardless of the number of corticosterone pulses administered, the baseline CA1 fEPSP response before high frequency stimulation was not acutely affected.

**Table 4:** Averaged values of the baseline CA1 fEPSP slope (mV/ms) of hippocampal brain sections of young adult mice that have been exposed to either four corticosterone or vehicle pulses (P1-4). For each pulse the average baseline is indicated before (aCSF) and during corticosterone exposure. The values are expressed as mean ± SEM for each experimental group (based on 6–9 recordings per group).

Baseline slope during infusion (mV/ms)	P1	P2	P3	P4
**Corticosterone**				
***aCSF***	538 ± 80	439 ± 70	498 ± 70	373 ± 31
***Corticosterone***	579 ± 94	449 ± 75	519 ± 88	372 ± 33
**Vehicle**				
***aCSF***	418 ± 32	481 ± 49	482 ± 77	509 ± 76
***Corticosterone***	431 ± 36	482 ± 55	474 ± 71	511 ± 76

### The effect of corticosterone pulses on synaptic plasticity

We have previously shown that high frequency stimulation (HFS) at the end of a (single) pulse of corticosterone enhances the degree to which LTP can be evoked when compared to vehicle treatment [24]. Here, we examined how and if synaptic plasticity was also affected during subsequent corticosterone pulses. HFS coincided with the end of the corticosterone administration period and was thus applied at the peak of each corticosterone pulse.

In line with earlier findings [24], clear LTP was seen when high frequency stimulation was applied at the end of the first pulse of corticosterone (Fig 3B; p < 0.01 compared to the pre-HFS baseline) and the degree of LTP was more pronounced than seen in the vehicle group (Fig 3C; p < 0.01 vs vehicle). However, during subsequent corticosterone pulses less to no synaptic potentiation could be evoked: when corticosterone was given at the peak of the 3^rd^ or 4^th^ pulse, no LTP was seen in response to HFS (p = 0.76 and p = 0.30, compared to the pre-HFS baseline respectively). This was supported by a two-way ANOVA that showed a significant interaction effect (pulses x treatment; F_(3, 50)_ = 4.67; p < 0.01). In contrast, slices treated with vehicle pulses all showed highly comparable, significant LTP after HFS stimulation, even after 4 vehicle treatments (Fig 3C; p values ranging from < 0.001 to < 0.01). These latter results suggest that the corticosterone-induced attenuation in LTP in response to HFS during the 4^th^ corticosterone pulse was not due to a general rundown of the slices or inability to induce LTP.

## Discussion

Biomathematical modelling studies have elegantly demonstrated that glucocorticoid pulsatility is generated and maintained via rapid, ultrashort feedforward and feedbackward cycles between the adrenal and pituitary gland which are reliably maintained across the blood-brain barrier [10,29]. These continuous cycles essentially serve to maintain homeostasis and provide a self-regulatory mechanism that allows rapid adaptation in the face of acute stress, including full transcriptional responses to corticosterone [1,30]. Over the past decade it has been realized that limbic cells are also rapidly and transiently altered in excitability through rapid, non-genomic actions of corticosteroids [18]. These studies however did not consider the physiological patterned nature of steroid release. In the current study we examined whether administration of multiple corticosterone pulses to hippocampal cell cultures or slices (with an interval relevant for ultradian rhythmicity) repeatedly changes hippocampal excitability, potentially changing the information flow through this area. The main finding is that hippocampal CA1 cells reliably ‘translate’ the first shift in corticosteroid levels into altered glutamate transmission and LTP. However, eventually the corticosterone-induced changes become more variable, attenuated or even reversed (as in the case of LTP), mitigating the potential changes in excitability *during* the pulses. Based on these findings, we would argue that at the start of the active phase hippocampal excitability is potentially more extensively changed by the first high-amplitude pulse than during later pulses. This complements earlier studies suggesting that baseline glutamatergic transmission (i.e. in-between pulses) possibly is also most markedly affected after the first pulse, but that subsequent pulses help to restore baseline glutamatergic transmission to the situation prior to the first large-amplitude pulse [17].

The currently used paradigm of repeated hourly corticosterone exposure is reminiscent of the protocol used in earlier studies by others, thereby increasing generalizability [9,17,31,32]. Yet, it should be emphasized that this design only partly mimics the natural situation where the amplitude of the ultradian pulses shows gradual circadian modulation according to the time of day. In that respect it could be argued that the current design represents a reduced model as it uses multiple, identical dosages of relatively high concentrations (100 nM). Future studies that consider both time and amplitude aspects simultaneously are therefore recommended. This is a very relevant issue since gradual increases (or decreases) in pulse amplitude may affect excitability differently than a sequence of high amplitude pulses on a low corticosterone background, as presently tested. In addition, the response to pulses of corticosterone under the here applied in vitro conditions, where e.g. GABAergic transmission is blocked and hippocampal tissue is artificially depleted from its natural inputs, may differ from that seen in vivo. The extrapolation of our findings to functional consequences of corticosterone pulsatility for glutamate transmission and synaptic plasticity–at the start of the active phase- in freely moving animals therefore needs to be done with care.

Previous studies have shown rapid alterations in mEPSC frequency, GluA2-AMPAR surface dynamics and synaptic plasticity during a single corticosterone application (reviewed in [18]). We fully replicated these earlier findings. The stability of responses to subsequent corticosterone pulses–though generally attenuating over time- appears to diverge somewhat, depending on the experimental parameter. In both the AMPA receptor surface trafficking and LTP experiments we observed a gradual decline or even reversal of responses (in the case of LTP) during the later pulses. These parameters were not overall compromised due to repeated tissue manipulations and/or the progression of time, because we saw no attenuation in these output parameters during the 4^th^ pulse of a vehicle. Rather, the inability to keep up the effects seen in response to the 1^st^ pulse after a succession of corticosterone pulses appears to be a reproducible phenomenon observed with different output parameters, tissues and experimental approaches. Interestingly, spontaneous glutamate release, as measured via mEPSC frequency, was also attenuated during the 3^rd^ pulse but seemed to partially recover during the next application. We have no explanation for this phenomenon. Yet, this recovery was another piece of evidence that the tissue is still able to respond to corticosterone, and that attenuation is not a mere artifact or reflects overall deterioration of the tissue. We cannot exclude, though, that the absence of corticosterone pulses during the time that slices are prepared and incubated (prior to in vtiro corticosterone application) may amplify the response to the first pulse, compared to the later ones.

Although we did not aim to address the mechanism underlying the corticosteroid actions, the time frame of our experimental protocol (e.g. recordings *during* corticosterone pulses) suggests the involvement of non-genomic pathways. Earlier studies on rapid corticosterone effects in the hippocampus pointed to a role of putative membrane-MRs [19,21,22,25]. Yet, the timeframe of the attenuation in the various parameters (1–3 hrs) is in line with genomic events and could indicate interaction between rapid and slow effects of corticosteroids [33]. Possibly, attenuation in rapid corticosteroid actions involves internalization of MRs, similar to what has been described for other receptors [34,35]. However, we can certainly not exclude that the attenuation seen with later corticosterone pulses is caused by other gradually developing genomic effects initiated during the first pulse(s). In accordance, previous large-scale microarray studies have shown that the 1–3 hrs delay between the 1^st^ pulses and later pulses (when attenuation becomes discernable) is a critical time-domain in which genomic effects start to develop [33], and coincides with consolidation of stress-related information [18]. The delineation of such complex interactions between rapid and delayed corticosteroid effects awaits further investigation. We did recently provide the first evidence–in BLA neurons- that a first corticosterone pulse can indeed interact with and determine the outcome of the second pulse, regarding neuronal excitability and synaptic plasticity, and that this relies on a GR-dependent pathway [25]. Moreover, in the CA1 region, the baseline glutamatergic response 2h after a 1^st^ pulse appeared to be affected by the absence or presence of a 2^nd^ pulse applied approximately 1h after the first; this also supports a complex interaction between rapid and delayed responses to corticosterone in the face of multiple pulses [17]. Of note, this earlier study did not address what happens *during* transient elevations in corticosterone level, which was the subject of the present study.

In general, our study indicates that transient elevations of corticosterone–such as may happen during ultradian pulses- initially promote aspects of hippocampal glutamatergic transmission and synaptic plasticity. However, large-scale changes in hippocampal excitability due to a sequence of ultradian corticosterone pulses are less likely to occur, at least not with respect to spontaneous release of glutamate-containing vesicles, GluA2-AMPAR surface dynamics or evoked field responses; on the contrary, a brief reduction in the ability to evoke LTP at the peak of ultradian pulses appears to develop over time. As mentioned, to what extent the present findings depend on the dynamics of the experimental paradigm will need to be examined in future studies. For the moment, the findings on spontaneous glutamate transmission and GluA2 mobility are compatible with the view that beneficial effects of ultradian pulses on limbic transcriptional regulation [14–17] do not necessarily come at the cost of hourly fluctuations in background excitability. We tentatively suggest that in this respect the observed phenomenon may keep hippocampal neurons optimally responsive to incoming signals related to environmental challenges.

## Abbreviations

HPA: hypothalamic-pituitary-adrenal
GR: glucocorticoid receptor
EPSC: excitatory postsynaptic current
LTP: long-term potentiation
MR: mineralocorticoid receptor
